# Neuroprotection mediated by cystatin C-loaded extracellular vesicles

**DOI:** 10.1038/s41598-019-47524-7

**Published:** 2019-07-31

**Authors:** Rocío Pérez-González, Susmita Sahoo, Sebastien A. Gauthier, Yohan Kim, Meihua Li, Asok Kumar, Monika Pawlik, Luisa Benussi, Roberta Ghidoni, Efrat Levy

**Affiliations:** 1 Nathan S. Kline Institute, Orangeburg, NY USA; 2 Department of Psychiatry, NYU Langone Medical Center, New York, NY USA; 3 Molecular Markers Laboratory, IRCCS Istituto Centro San Giovanni di Dio-Fatebenefratelli, Brescia, Italy; 4 Department of Biochemistry and Molecular Pharmacology, NYU Langone Medical Center, New York, NY USA; 5 The Neuroscience Institute, NYU Langone Medical Center, New York, NY USA; 6 Present Address: Center for Networked Biomedical Research on Neurodegenerative Diseases (CIBERNED), Department of Neurology, Hospital de la Santa Creu i Sant Pau, Barcelona, Spain; 7 Present Address: Cardiovascular Research Center, Icahn School of Medicine at Mount Sinai, NY New York, USA

**Keywords:** Exocytosis, Stress and resilience

## Abstract

Cystatin C (CysC) is implicated in neuroprotection and repair in the nervous system in response to diverse neurotoxic conditions. In addition to being secreted from cells in a soluble form, CysC is released by cells in association with extracellular vesicles (EVs), including exosomes. We demonstrate that EVs containing CysC protect cultured cells from starvation-induced death. Moreover, while EVs secreted by CysC-deficient cells were not protective, EVs secreted by CysC-deficient cells treated with exogenous human CysC significantly enhanced the survival of the cells. CysC also plays a role in modulating the secretion of EVs, enhancing secretion of EVs by primary cortical neurons and primary cortical smooth muscle cells. Confirming these *in vitro* findings, higher EV levels were observed in the brain extracellular space of transgenic mice expressing human CysC as compared to littermate controls. Regulation of cell-secreted EV levels and content in the brain is likely to be essential to maintaining normal brain function. We propose that enhanced EV release could rescue the deleterious effects of dysfunction of the endosomal-lysosomal system in neurodegenerative disorders. Moreover, a higher level of CysC-loaded EVs released from cells in the central nervous system has important protective functions, representing a potential therapeutic tool for disorders of the central nervous system.

## Introduction

CysC is a ubiquitously expressed secreted protein that is abundant in brain tissue, where it is expressed by neurons, astrocytes, microglia, and vascular endothelial and smooth muscle cells (SMC) [reviewed in^1^]. CysC was originally identified as an inhibitor of cysteine proteases, yet recent data are consistent with CysC playing diverse roles in the response of cells to injury and stress, where mobilization of CysC appears to alleviate specific pathological consequences [reviewed in^1,2^]. CysC has been suggested to play a role in nervous system repair following injury and disease [reviewed in^1,3^], and its levels are altered in the brain, in the cerebrospinal fluid (CSF) and within specific neuronal populations in multiple neurodegenerative diseases^4–18^. Accumulated data suggest that increased CysC cellular expression in the brain is part of a neuroprotective response aimed at preventing or minimizing neurodegeneration [reviewed in^2^].

Newly synthesized CysC is secreted from all cell types^19–24^, secreted CysC can be internalized into cells via endocytosis^25–27^, and uptake of CysC can occur by cells other than the cell producing the protein^25–27^, suggesting that *in vivo* CysC can mediate important communications between cells. In addition to being targeted to the classical secretory pathway, CysC is secreted in association with EVs^28^. EVs are phospholipid bilayer membrane-enclosed vesicles that contain lipids, proteins, and RNA, that are released by cells into tissue extracellular space, biological fluids, and conditioned cultured media. Various species of EVs have different intracellular origin [reviewed in^29–31^]. While microvesicles derive from the plasma membrane, exosomes are generated as intraluminal vesicles (ILVs) in late-endosomes/multivesicular bodies (MVBs) in a process that can be mediated by either the endosomal sorting complexes required for transport (ESCRT) machinery, which includes TSG101 and its associated protein Alix, or by ESCRT independent systems that include tetraspanin proteins^30^. The formation of ILVs involves the sequestration of proteins, lipids, and other cellular components that are targeted either for degradation in lysosomes or for release into the extracellular space in association with exosomes [reviewed in^32,33^]. Secreted exosomes are uptaken and internalized by recipient cells [reviewed in^34^]. Thus, exosome secretion may be an additional mechanism for the cell-to-cell transfer and propagation of CysC-mediated protection.

We have previously demonstrated a concentration-dependent protective effect of CysC on neuronal cell lines, primary cortical neurons, and primary cortical SMC from death induced by various toxic factors^35,36^, and an Alzheimer’s disease (AD) associated impairment of the release of exosomal CysC^28^. The studies described here reveal that CysC-containing exosome-enriched EVs protect cells from starvation-induced death. Moreover, we show that CysC regulates secretion of EVs and that the secreted EVs can be loaded with CysC. These data suggest a double protective role of CysC-associated EVs because of the benefit of EV release via the endocytic pathway coupled with the benefits of CysC.

## Results

### *In vitro* loading of EVs with CysC

**Figure 1:**
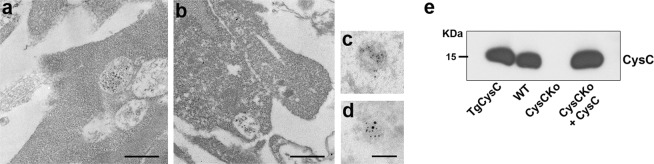
Loading of EVs with CysC. MVBs in CysCko SMC treated with 0.15 μM CysC contain intraluminal vesicles immuno-labeled with an antibody to CysC (**a**,**b**). EVs secreted by SMC not-treated (**c**) and CysC-treated (**d**) were immuno-labeled with antibodies to CysC (10 nm gold particles) and TSG101 (6 nm gold particles). Bars 500 nm (**a**,**b**), 100 nm (**c**,**d**). Western blot showing the content of CysC in EVs secreted by SMC isolated from brains of TgCysC, wild-type, CysCko mice and CysC-treated CysCko cells (**e**).

We investigated whether exogenous CysC is internalized into MVBs, the source of exosomes, and whether this internalized CysC is then associated with exosomes. Full-length human CysC was added to the medium of cultured cells isolated from the brains of CysC knockout mice [CysCko^37^] in order to uniquely detect internalized exogenous CysC by immuno-electron microscopy (immuno-EM). We used primary cortical SMC because they are both larger than and have higher levels of endocytosis compared to primary cortical neurons (data not shown). Immuno-EM revealed that CysC was endocytosed and seen in association with ILVs within MVBs (Fig. 1a). Additionally, immuno-EM showed CysC in association with ILVs in the process of being released from the cell upon fusion of the MVBs with the plasma membrane (Fig. 1b).

*In vitro* studies have shown that cells release subpopulations of EVs with distinct molecular and biological properties^38,39^. These include plasma membrane-derived microvesicles^40^ and exosomes formed by the invagination of the membrane of late endosome/MVBs around cytoplasmic materials [reviewed in^41,42^]. In order to determine whether exogenously applied CysC is secreted in association with exosomes, whole-mount immuno-EM was conducted using dual staining with antibodies to the exosomal marker TSG101 (smaller 6 nm nanogold) and to CysC (larger 10 nm nanogold) in EVs isolated from both CysCko and CysC-treated CysCko cells. EVs isolated from both CysCko and CysC-treated CysCko cells were positive for TSG101 demonstrating secretion of exosomes from both cell types. While no CysC staining was found in EVs secreted by cells isolated from CysCko mice (Fig. 1c), anti-CysC antibody labeled exosomes secreted by CysC-treated CysCko cells (Fig. 1d). Western blot analysis confirmed that cells isolated from the brain of transgenic mice expressing human CysC (TgCysC) and wild-type mice, as well as CysC-treated CysCko cells, secret CysC in association with EVs (Fig. 1e). Importantly, these data show that exogenously applied CysC is secreted by cells in association with EVs.

### Uptake of exogenous CysC in association with EVs by recipient cells

**Figure 2:**
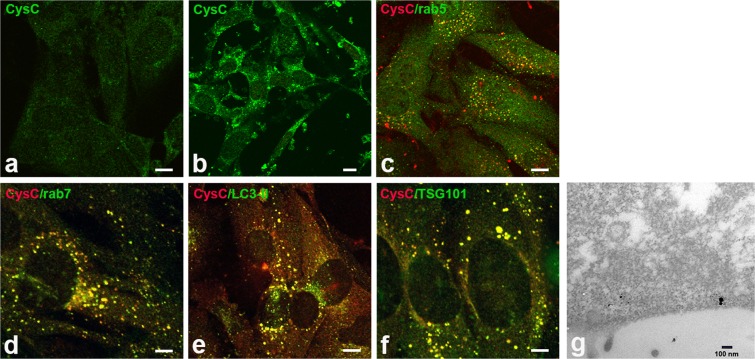
Endocytic uptake of EVs by SMC isolated from CysCko mice. Immunocytochemical staining with anti-CysC antibodies: EVs from SMC isolated from CysCko mice, incubated in the absence (**a**) or presence of 0.15 µM CysC (**b**), EVs applied for 1 hr. Immunocytochemical staining with antibodies to CysC (red) and rab5, a marker of early endosomes (green), EVs applied for 20 min (**c**), rab7, a marker of late endosomes (green), EVs applied for 1 hr (**d**), LC3-II, a marker of autophagic vacuoles (green), EVs applied for 16 hrs (**e**), and TSG101, a marker of exosomes (green), EVs applied for 1 hr (**f**). Electron micrograph of uptake of EVs isolated from CysCko mice, incubated with CysC, by SMC isolated from a CysCko mouse, EVs were applied for 20 min. Dual staining with anti-CysC (6 nm gold particles) and anti-TSG101 (10 nm gold particles) (**g**). Bars 10 μm (**a**–**c**,**e**), 5 μm (**d**,**f**) and 100 nm (**g**).

Isolation of vesicles from culture media *in vitro* and from brain extracellular space *in vivo* yield small EVs (smaller than 200 nm). Most *in vitro* studies described here used SMC as a ready source for EVs isolated from the growth media. EVs were isolated from serum-free media of SMC derived from brains of CysCko mice, either in the absence of or exogenously supplemented human CysC. Isolated EVs from both conditions were applied to SMC isolated from CysCko mice cultured in serum-free medium, and their uptake was examined by immunocytochemistry and immuno-EM using antibodies to endosomal or exosomal markers together with an anti-CysC antibody. When CysCko SMC-derived EVs were applied to the culture media of CysCko SMC, no CysC staining was detected in the EVs-treated cells (Fig. 2a). When CysC-supplemented-CysCko SMC-derived EVs were applied to the culture media of CysCko SMC, CysC staining was detected within the EV-treated cells (Fig. 2b). These data show that exogenously-applied CysC was endocytosed by treated CysCko cells and secreted via EVs that were uptaken by the CysC-containing EV-treated CysCko cells. Endocytosis of the EVs via the endosomal-lysosomal pathway as well as their uptake into autophagic vacuoles was demonstrated by staining with antibodies to specific markers of the various compartments (Fig. 2c–e). The uptake of CysC in association with exosomes was demonstrated by double immunostaining with antibodies to CysC and the exosomal marker TSG101 (Fig. 2f). Uptake of CysC in association with exosomes was also demonstrated by immuno-EM with antibodies to CysC and TSG101 of CysCko SMC treated with EVs that were isolated from media of CysC treated cells (Fig. 2g). The results show that CysC is uptaken in association with exosomes by recipient cells by endocytosis.

### EVs containing CysC protect primary cortical SMC and primary neurons from nutrition deprivation

**Figure 3:**
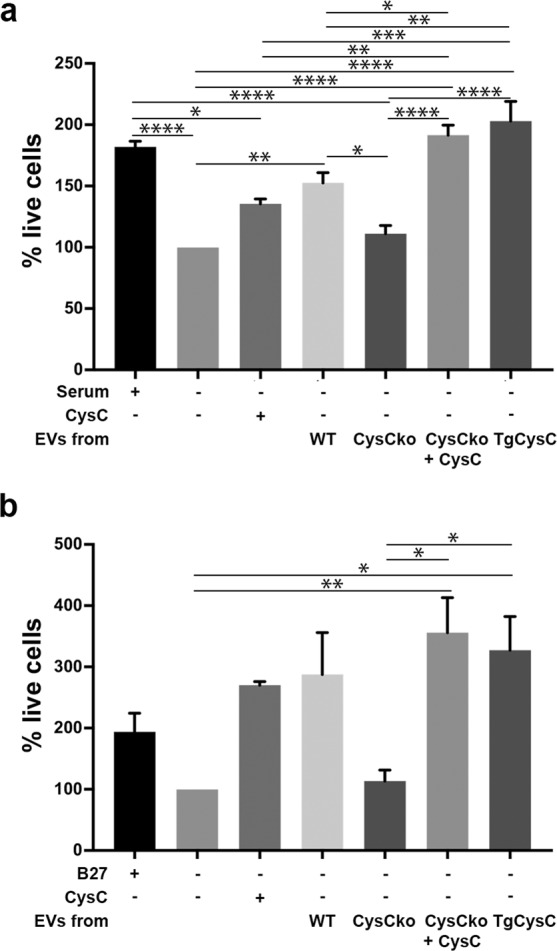
EVs containing CysC protect primary cortical SMC and primary cortical neurons isolated from CysCko mice. EVs secreted by primary cortical SMC from wild-type (WT), CysCko, CysCko treated with CysC, and TgCysC mice, were isolated and equal volume of EVs was added to the cultures of either primary cortical SMC (**a**) or neurons (**b**). Full-length human CysC protein, not associated with EVs was added as a positive control. Data normalized relative to cells in medium without serum (**a**) or without B27 (**b**). Mean and SEM are presented (n = 4; *p < 0.05, **p < 0.01, ***p < 0.001, ****p < 0.0001).

Our previous data showed that exogenous human CysC added to the growth media protects cultured cells from death, including from nutrition deprivation and growth supplement deprivation^35^. Therefore, we tested the effect of CysC in association with EVs on cells under similar deprivation conditions. Nutrition starvation-stress was induced in primary cortical SMC or neurons isolated from CysCko mice by withdrawing serum supplement or the growth supplement B27, respectively. EVs were isolated from the culture media of SMC derived from either wild-type, TgCysC, or CysCko mice, and from CysCko cells treated with full-length human CysC protein. These EVs with different levels of CysC were added to primary cortical SMC or neurons under starvation. Full-length human CysC protein was added to the cultures as a control for the effect of soluble CysC, not associated with EVs. Live cells were quantified by the MTS assay. Our data showed that EVs from either wild-type or TgCysC cells protected the recipient CysCko cells from starvation-induced death, whereas EVs from CysCko cells did not (Fig. 3a,b). In addition, EVs from CysCko SMCs, treated with exogenous human CysC significantly enhanced the survival of the CysCko SMCs (Fig. 3a) or primary neurons (Fig. 3b) treated with these EVs. These data demonstrate that CysC secreted in association with EVs is protective.

### Regulation of EV secretion by CysC

**Figure 4:**
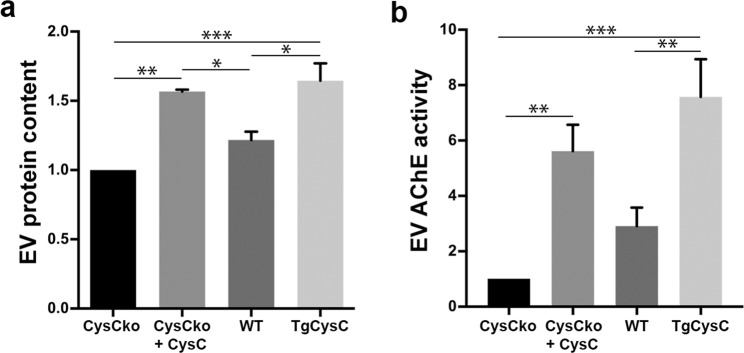
CysC regulates the level of EV secretion. EVs were isolated from conditioned media of SMC isolated from wild-type (WT), TgCysC, and CysCko mice, and of CysCko cells incubated in the presence of 0.15 µM CysC for 24 hrs. EV protein content (**a**) and EV AChE activity (**b**) were standardized relative to protein amount in the cell lysates. Data were normalized to levels of EV proteins and AChE activity, respectively, in EV samples isolated from conditioned media of CysCKo SMC, presented as mean ± SEM (n = 8; *p < 0.05, **p < 0.01, ***p < 0.001).

**Figure 5:**
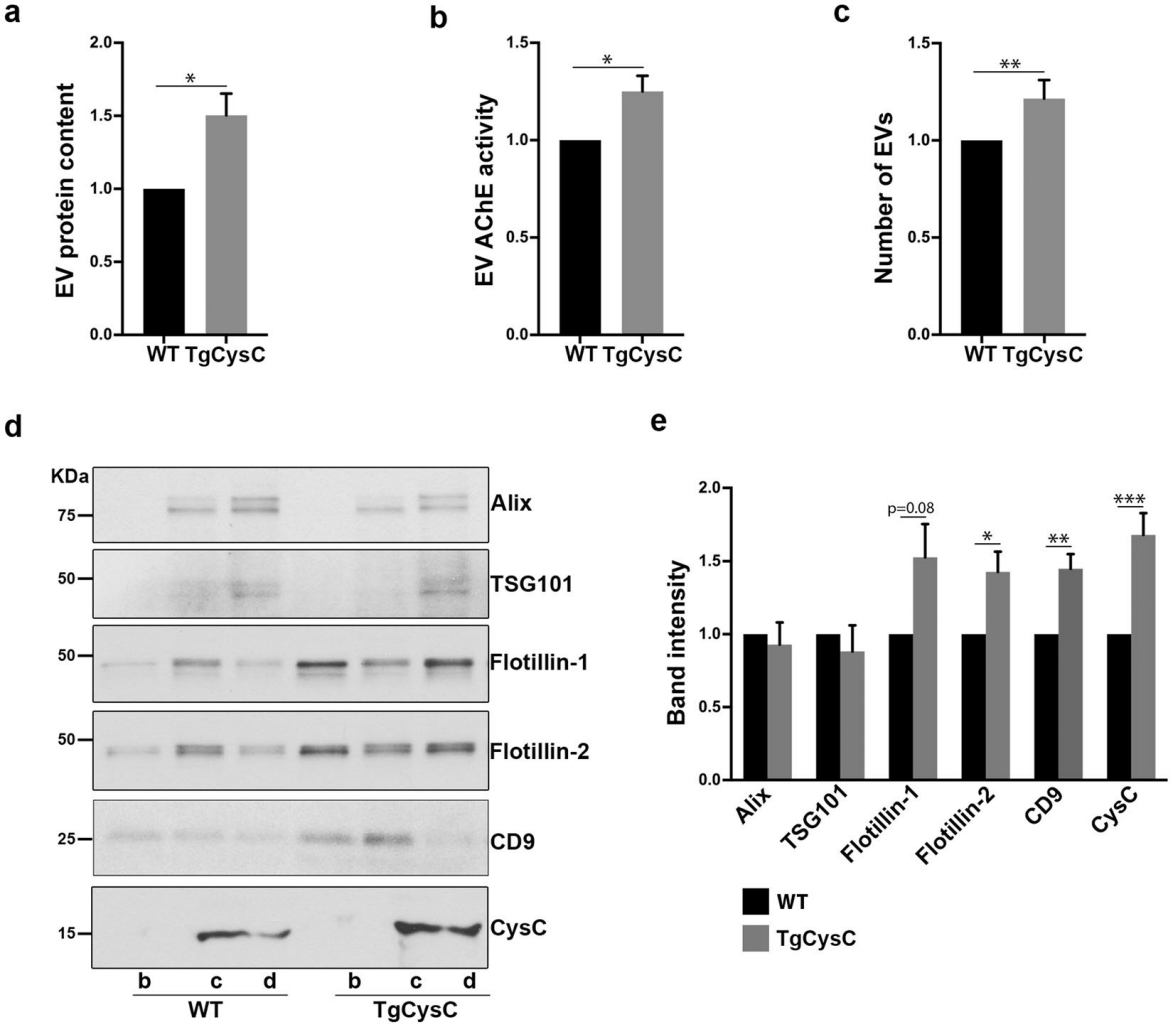
CysC enhances EV levels in the brain extracellular space of CysC transgenic mice but does not specifically regulate ESCRT-dependent exosome secretion. EVs were isolated from hemibrains of 12-month-old TgCysC and wild-type (WT) littermate control mice. EV protein content (**a**), EV AChE activity (**b**) and the number of particles in the EV brain preparations assessed by NTA as described in the Methods (**c**) were standardized relative to protein amount in the hemibrain. Representative Western blots of the sucrose-gradient fractions b, c and d containing EVs (**d**) and the corresponding quantification (**e**) showing the levels for the EV markers Alix, TSG101, CD9, Flotillin-1, and Flotillin-2 in EVs isolated from the brains of TgCysC and WT mice. Elevated CysC levels in TgCysC EVs due to the CysC overexpression in the transgenic mice are also shown. All data are shown as the TgCysC to control ratio and are presented as mean and SEM (*p < 0.05; **p < 0.01).

Unexpectedly, quantification of EVs secreted by cells in the experiments described above consistently identified CysC as a previously unknown regulator of EV secretion. We measured the levels of EV secretion by quantifying the ratio of EV proteins to the total protein amount in cell lysates and by quantifying acetylcholinesterase (AChE) enzyme activity, a protein that is specifically sorted into EVs^43^. SMC derived from TgCysC mice secreted significantly more EVs than cells derived from brains of wild-type mice, and cells from CysCko mice secreted a low number of EVs (Fig. 4). Moreover, treatment of cells derived from CysCko mice with CysC greatly enhanced EV secretion (Fig. 4). Additionally, we explored EV secretion *in vivo* in the brain of TgCysC mice and littermate wild-type controls. Consistent with our *in vitro* finding, significantly higher total EV protein (Fig. 5a) as well as significantly higher AChE enzyme activity (Fig. 5b) were observed in the EVs isolated from the brain of TgCysC mice as compared to controls. Further, supporting the protein and AChE data, nanoparticle tracking analysis (NTA) of the EV preparations revealed higher number of vesicles in the brain of TgCysC mice (3.87 × 10^10^ ± 0.18 particles/mg of tissue) compared to controls (3.19 × 10^10^ ± 0.12 particles/mg of tissue) (Fig. 5c). Collectively, these *in vitro* and *in vivo* data show that CysC enhances EV secretion. In order to investigate which subpopulation of small EVs was differently secreted in the brain of TgCysC mice compared to wild-type controls, we performed Western blot analysis (Fig. 5d). No difference in the level of the exosomal proteins TSG101 and Alix was observed between TgCysC and wild-type littermate control mice (Fig. 5e). However, when we analyzed the levels of Flotillin-1 and Flotillin-2, lipid rafts proteins present in all EV subtypes^44^, and CD9, one of the most abundant tetraspanins found in EVs^44^, we detected a significant increase in TgCysC brain EVs compared to controls (Fig. 5e). These data suggest that while CysC enhances EV release, it does not specifically affect the secretion of exosomes generated by the ESCRT machinery. Instead, other small EVs such as exosomes generated by ESCRT-independent mechanisms or plasma membrane-derived microvesicles are affected by CysC overexpression.

## Discussion

Multiple studies have shown that CysC is protective during the response of cells to injury and stress by diverse mechanisms, including the inhibition of cysteine proteases, the induction of autophagy, and the induction of cell proliferation [reviewed in^1,2^]. CysC protects cultured neurons by inducing functional autophagy via the mTOR pathway, including competent proteolytic clearance of autophagy substrates by lysosomes^35^. *In vivo* studies have shown that exogenous CysC ameliorates the extent of early brain injury and learning deficits in experimental subarachnoid hemorrhage in rats, possibly through activating the autophagy pathway^45^. CysC also has anti-aggregation properties, inhibiting amyloid β aggregation into fibrils and oligomers^46–49^, which is consistent with genetic data showing a linkage of a CysC gene polymorphism that reduces cellular CysC secretion with an increased risk of developing late-onset AD^1,50–52^. Moreover, a low level of transgene CysC overexpression alleviates multiple pathologies observed in a mouse model of Down syndrome, including neuronal endosomal alterations and behavioral deficits^53^. Here we demonstrate a previously unknown protective mechanism modulated by CysC levels: the protection of brain cells by CysC-loaded EVs amplified by enhanced CysC-induced EV release. We have previously demonstrated an interrelationship between the endosomal and exosomal pathways, suggesting that EV release compensates for the endosomal abnormalities seen in early stages of sporadic AD, familial AD, and Down syndrome^54–56^, and that a decrease in exosome release contributes to endosomal dysfunction^55,57^. Thus, we hypothesized that in compensation for a dysfunction of the endosomal-autophagic-lysosomal pathway, neurons may enhance the release of intracellularly accumulated material via EVs. Supporting this, we found that exosome secretion is enhanced in the brains of Down syndrome patients and a mouse model of the disease. In the mouse model, the increase in exosome release occurred at a later age than the appearance of endosomal pathology, suggesting that enhanced exosome secretion in Down syndrome aims to shed more endosomal content into the brain extracellular space by neurons with enlarged endosomes^56^. A similar mechanism of exosome release was suggested for Niemann-Pick Type C disease to release free cholesterol that accumulates within late endosomes and lysosomes^58^. In human post-mortem tissue and mouse models humanized for apolipoprotein E, we have examined the impact of apolipoprotein E4, the greatest genetic risk factor for AD, on brain exosomes. Compared to humans or mice homozygous for the risk-neutral E3 allele we showed that the E4 allele drives lower exosome levels in the brain extracellular space. In mice, we show that the apolipoprotein E4-driven change in brain exosome levels is age dependent, occurring earlier than our previously reported brain endosomal pathway changes, arguing that an apolipoprotein E4-driven failure in exosome production plays a primary role in endosomal and lysosomal deficits^57^. Similar to the protective role of CysC-loaded EVs shown here, we have demonstrated in granulin-associated frontotemporal dementia that exosome release and composition is strongly impaired. Along with shortage of the circulating progranulin, a neurotrophic factor, a decrease in EV release contributes to progranulin deficiency^59^. We propose that high levels of CysC expression, as observed in neuronal population vulnerable to AD-related pathology^11,12^, serve to increase EV release. While exosome release from MVBs mediates the protective elimination of abnormally accumulated proteins, such as the amyloidogenic proteins amyloid β, tau, prion protein, and α synuclein from the endosomal-autophagic-lysosomal compartments, it was suggested that exosome release can propagate misfolded protein pathology within the brain [reviewed in^1,2^]. However, given that CysC has anti-aggregation properties, inhibiting amyloid β aggregation into fibrils and oligomers^46–49^, exosomal-associated CysC may have an anti-amyloidogenic function and mitigate the negative effects of the secretion of exosomes containing misfolded proteins.

Secreted exosomes can be taken-up by other cells, where their content can be delivered^60^. We demonstrate that while both CysC-containing and CysC-deficient EVs are taken up by cultured cells, only those containing CysC can protect cells from serum-deprivation- and supplement-deprivation-induced death. The physiological significance as well as therapeutic potential of exosomes is in part the result of their limiting membrane, which renders them more stable in the extracellular environment than soluble proteins [reviewed in^32^]. Therefore, the association of CysC with exosomes likely prolongs the extracellular survival of CysC, with extended ability to exert its protective effects. We propose a novel treatment and/or preventive intervention for neurodegenerative disorders based on administration of autologous exosomes released by dendritic cells and loaded with CysC. This therapy is based on the protective activity of exosomes containing and delivering the protective agent CysC to cells.

## Methods

### Mice

TgCysC transgenic mice express human CysC under the transcriptional control of its own promoter, expressing the transgene along with its endogenous counterpart in the appropriate tissues, both in the brain and the periphery^49,61^. Non-transgenic littermate mice were used as controls. CysC knockout (CysCko) mice were described by Huh *et al*.^37^. All animal procedures were performed following the National Institutes of Health guidelines with approval from the Institutional Animal Care and Use Committee at the Nathan S. Kline Institute for Psychiatric Research.

### Primary cultures of cortical neurons and cortical smooth muscle cells

Primary cultures of cortical neurons were established from cortices containing the hippocampus of prenatal E16 pups of pregnant wild-type (WT), CysCko, and homozygous TgCysC mice as we described^35^. Briefly, hippocampi and cortices were dissected in 20 units/ml papain (Worthington Biochemical, Lakewood, NJ) in Hibernate E (Brainbits, Springfield, IL) solution (3 ml per hemibrain) for 15 min at 37 °C. After trituration and settlement, the cell suspension was plated in 96-well dishes at a density of 250,000 cells/well. For immunocytochemistry and immuno-electron microscopy (Immuno-EM), cells were plated onto poly-ethyleneamine coated coverslips in 12-well culture plates at a density of 70,000 and 1.5 million cells/well, respectively. Cells were maintained in Neurobasal supplemented with 2% B27, 0.5 mM L-glutamine.

SMC were isolated from microvessels of 4–6 months old mouse brains as we described^62^. Briefly, the isolated brains were minced in ice-cold DMEM-HEPES, centrifuged at 300 × g for 10 min, the pellet was digested in 0.05% collagenase dispase in DMEM-HEPES for 30 min and centrifuged at 300 × g for 10 min. The resulting pellet was centrifuged at 10,000 × g for 10 min at 4 °C on a 17% dextran density gradient and the pellet resuspended in complete DMEM media (DMEM containing 10% FBS, 2 mM L-glutamine, 100 U/ml penicillin, 100 μg/ml streptomycin, 0.1 mM NEAA, and 50 μg/ml DNAse). The microvessels were screened through 2 mm glass beads. The isolated microvessels were digested in 0.05% collagenase-dispase diluted in complete DMEM media for about 3 hrs and plated in 12-well tissue culture plates.

When indicated, 0.15 μM of human urinary CysC (Calbiochem- EMD Bioscience, San Diego, CA) was added into the culture media.

### EV isolation from culture media

EVs were isolated from SMC media as previously described^63^ by serial centrifugations and ultracentrifugations. Briefly, serum-free media was added to 90% confluent cells grown in T75 flasks and collected after 24 hrs. 65 ml of conditioned media were sequentially centrifuged at 300 × g for 10 min at 4 °C, 2,000 × g for 10 min at 4 °C, and at 10,000 × g for 30 min at 4 °C to discard cells, membranes, and debris and larger EVs. The supernatant was centrifuged at 100,000 × g for 70 min at 4 °C to pellet EVs. The EV pellet was resuspended in cold phosphate buffer (PBS) (Invitrogen, Carlsbad, CA) and the EV solution was centrifuged at 100,000 × g for 70 min at 4 °C. The final EV pellet was resuspended in 20 µl of PBS. Two µl of the EVs in PBS were used for the AChE assay. The leftover solution was mixed with an equal volume of 2x radio-immune precipitation assay (RIPA) lysis buffer supplemented with a mixture of protease inhibitors. Two µl were used to quantify EVs protein content (BCA Protein Assay Kit; Pierce, Rockford, IL).

### EV isolation from mouse brains

EVs were isolated from frozen brains of heterozygous TgCysC and littermate wild-type controls as we have previously described^64,65^. Briefly, one mouse hemibrain was treated as described above for the isolation of primary cultures of cortical neurons. The brain tissue was gently homogenized in Hibernate A and the homogenate was sequentially filtered through a 40 μm mesh filter (BD, Franklin Lakes, NJ) and a 0.2 μm syringe filter (Corning, Corning, NY). EVs were isolated from the filtrate as described above^63^ and further purified on a sucrose step gradient as previously reported^64,65^. Sucrose gradient fraction pellets were resuspended in 20 μl of cold PBS. Two μl were used to measure AChE activity and the rest was homogenated in RIPA lysis buffer supplemented with protease inhibitors. Two µl of the lysate were used to quantify EV protein content (BCA Protein Assay Kit; Pierce, Rockford, IL).

### Immuno-electron microscopy (immuno-EM)

CysCko SMC were grown on collagen-coated coverslips overnight in complete medium, washed in PBS, and replaced with serum-free medium for additional 12 hrs. 10 μl of EVs isolated from SMC media were applied to the cells for different time periods, and the cells processed for immuno-EM. Cells were washed and fixed in 4% paraformaldehyde and 0.1% glutaraldehyde (Electron Microscopy Sciences, Hatfield, PA) in 0.1 mM sodium cacodylate buffer for 20 min at 37 °C followed by treatment with 0.1% sodium borohydride. Permeabilization was done with 0.05% triton X in PBS for 10 min, blocking with 1% BSA-c for 1 hr, and incubation in primary antibody diluted with 0.2% BSA-c in PBS for 6 hrs at room temperature and secondary gold conjugates diluted in 0.2% BSA-c for 4 hrs. Postfixation was done in 2.5% glutaraldehyde for 1 hr, and silver enhancement using 80 μl of R-gent SE-EM mixture (Aurion) for 1 hr in dark. After treatment with 0.5% osmium tetroxide in PBS for 15 min, serial dehydration was done with alcohol, cells were embedded in Epon 812 Mixture (Electron Microscopy Sciences), ultra-thin sections were cut, and stained with uranyl acetate.

Whole mount EM analysis of 2 µl of EV solutions fixed in 2% paraformaldehyde and 0.1% glutaraldehyde followed by methyl cellulose (2%)-uranyl acetate solution was done as previously described^63^. Immuno-gold labeling was performed using antibodies to TSG101 (Abcam, Cambridge, MA) and CysC (EMD Millipore, Billerica, MA). Cell sections and EVs were examined using Philips CM 10 electron microscope. Images were captured on a digital camera (Hamamatsu; model C4742-95) using Advantage CCD Camera System software (Advanced Microscopy Techniques Corporation).

### Acetylcholine esterase activity assay

AChE activity assay is based on the Ellman assay described previously^66^. Briefly, 2 µl of EVs suspended in PBS were diluted in 298 µl of the AChE assay working solution [1.25 mM Acetylthiocholine (A5751, Sigma-Aldrich, St. Louis, MO), 0.1 mM 5,5′-Dithio-bis(2-nitrobenzoic acid) (DTNB, D8130, Sigma-Aldrich) in 0.1 M PBS at pH 8.0] and incubated at 37 °C in the dark for 30 min. Optical density (OD) was measured at 412 nm to quantify AChE activity in the EVs solution. The values of and correlation between the EV protein content and AChE activity are presented in Supplementary Fig. 1.

### Nanoparticle tracking analysis

EVs isolated from brains of TgCysC and littermate wild-type controls were resuspended in PBS and quantified using the Zetaview Multiple Parameter Particle Tracking analyzer Classic S model equipped with a 488 nm laser. Prior to each analysis, the machine underwent a quality control analysis using deionized distilled water, followed by assessment of microparticles or latex beads of 100 nm size (ThermoFisher Scientific catalog number 5010 A) with an expected range of 100 nm ± 3.9 nm median and mean. Using a monochromatic laser beam at 488 nm, the suspension of EVs was visualized and videotaped at 30 frames/s in each of 11 video locations on the machine. All the acquisition settings (dilution factor: 40000, sensitivity: 65, maximum area: 1000, minimum area: 10 and minimum brightness: 10) were optimized and kept constant between samples. Each video taken was then analyzed to obtain a calculated concentration of vesicles.

### Western blot analysis

EV proteins were separated by 4–20% tris-glycine gel electrophoresis (Criterion precast gel, Bio-Rad, Hercules, CA) as described^64,65^. The proteins were electrophoretically transferred onto PVDF membranes (Immobilon, EMD Millipore, Billerica, MA). Membranes were incubated with antibodies to CysC (1:1000, EMD Millipore, Billerica, MA), Alix (1:1000, EMD Millipore), TSG101 (1:1000, GeneTex, Irvine, CA), Flotillin-1 (1:1000, BD Biosciences, San Jose, CA), Flotillin-2 (1:1000, BD Biosciences), CD9 (1:1000, Santa Cruz Biotechnology, Santa Cruz, CA), and Sec61B (1:1000, Proteintech, Rosemont, IL), a marker of endoplasmic reticulum as a negative control (data not shown). Protein bands were quantified using ImageJ software (NIH). Full-length blots are shown in Supplementary Fig. 2.

### Immunocytochemistry with isolated EVs

Isolated EVs were applied to the CysCko SMC to study the uptake of EVs. SMC were grown in serum-free medium for 6–8 hrs and EVs were added for different time periods. The cells were fixed with 4% paraformaldehyde at 37 °C for 20 min, blocked with 10% FCS in PBS for 1 hr, and incubated in antibodies to CysC, to intracellular compartments: rab5a (Santa Cruz Biotechnology, Santa Cruz, CA), rab7 (Sigma, St. Louis, MO), and LC3 (Novus Biologicals, Littleton, CO) or to the exosomal marker TSG101. The cells were visualized in a Zeiss inverted confocal microscope.

### Cellular protection assay

CysCko primary cortical neurons or primary SMC were incubated in either the presence or absence of growth-supplement B27 or serum, respectively. Equal volume (10 μl) of EVs or PBS were applied in duplicates to the cultured cells under B27 or serum withdrawal condition. The cells were incubated for 20 hrs at 37 °C and 5% CO_2_. Cellular viability assay was performed using the MTS reagent (Cell proliferation assay, Promega). Cellular survival was expressed as percentage of cellular survival in serum-free medium cultures. Mean and SEM were calculated for 4 separate experiments.

### Statistical analyses

The statistical significance was determined by one-way analysis of variance (ANOVA) followed by the Tukey’s *post hoc* test to determine significance of differences between multiple test groups. Student’s T-test was used to determine significance when only two groups were compared.

## Supplementary information

Supplementary data

## Data Availability

All data generated and analyzed during this study are included in this published article.
