# Supplementary material for: Neuroprotection mediated by cystatin C-loaded extracellular vesicles
Source: Sci Rep. 2019 Jul 31;9:11104. doi: 10.1038/s41598-019-47524-7 (PMC6668451; doi:10.1038/s41598-019-47524-7)
Supplement: Supplementary file 1 — Supplementary data [file 41598_2019_47524_MOESM1_ESM.pdf]

Neuroprotection mediated by cystatin C-loaded extracellular vesicles

Rocío Pérez-González, Susmita Sahoo, Sebastien A. Gauthier, Yohan Kim, Meihua Li, Asok Kumar, Monika Pawlik, Luisa Benussi, Roberta Ghidoni, and Efrat Levy

a

	**CysCko**	**CysCko + CysC**	**WT**	**TgCysC**
**EV protein (µg/µl) ± SEM**	0.73 ± 0.04	1.14 ± 0.06	0.88 ± 0.05	1.20 ± 0.1
**EV AChE activity (µU/µg cell protein) ± SEM**	40.7 ± 5.73	186.45 ± 7.70	100 ± 9.68	250.02 ± 16.08

b

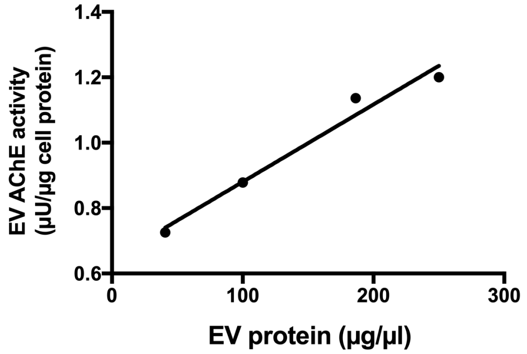

Supplementary Fig. 1. Quantitative data of EVs isolated from the conditioned media of SMC cells. Summary table showing the values (**a**) and correlation between EV protein content and EV AChE activity, determined by BCA and the AChE activity assay, respectively (R square= 0.9726) (**b**). U=Units.

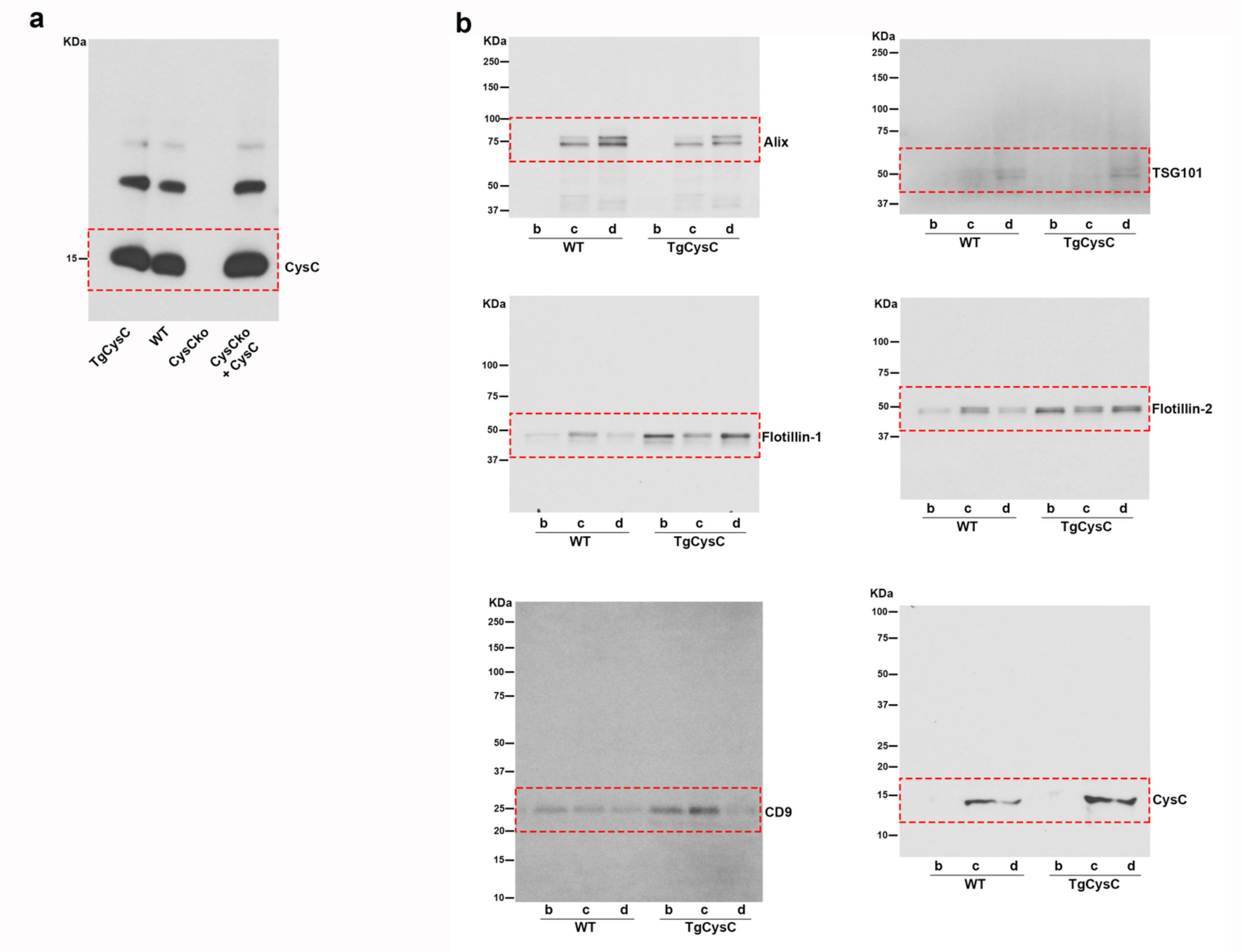

Supplementary Fig. 2. The full-length blots for the figures. a) Full-length blot for Fig. 1e. The upper bands correspond to dimers and trimers of CysC. **b**) Full-length blots for Fig. 5c. Note that the blots for Alix and TSG101 were cut below the 37 KDa protein-ladder band. The red dashed lines indicate the place where the images were cut to make the figures.
